# The Clinical Application of Platelet-Rich Plasma in the Female Reproductive System: A Narrative Review

**DOI:** 10.3390/life13122348

**Published:** 2023-12-15

**Authors:** Saaliha Vali, Srdjan Saso, Timothy Bracewell Milnes, James Nicopoullos, Meen-Yau Thum, James Richard Smith, Benjamin P. Jones

**Affiliations:** 1 Hammersmith Hospital, Imperial College NHS Trust, London W12 OHS, UK; srdjan.saso01@imperial.ac.uk (S.S.); jrsmithgynaecology@googlemail.com (J.R.S.); benjamin.jones@nhs.net (B.P.J.); 2 Department of Surgery and Cancer, Imperial College London, London W12 0NN, UK; 3 Department of Metabolism, Digestion and Reproduction, Imperial College London, London W12 0NN, UK; timothy.bracewell@imperial.ac.uk (T.B.M.); jamesn@lfclinic.com (J.N.); yauthum@lfclinic.com (M.-Y.T.); 4 Lister Fertility Clinic, The Lister Hospital, London SW1W 8RH, UK

**Keywords:** gynaecology, platelet-rich plasma, infertility, endometrium, uterus

## Abstract

Platelet-rich plasma is an autologous plasma containing platelets prepared from fresh whole blood drawn from a peripheral vein. Through processing, it can be prepared to contain supraphysiologic levels of platelets at three to five times greater than the level of normal plasma. PRP has been explored both in vivo and ex vivo in the human endometrium model in its ability to harness the intrinsic regenerative capacity of the endometrium. Intrauterine autologous PRP infusions have been shown to increase endometrial thickness and reduce the rate of intrauterine adhesions. In the setting of recurrent implantation failure, intrauterine infusion of PRP has been shown to increase clinical pregnancy rate. PRP also appears to hold a potential role in select patients with premature ovarian insufficiency, poor ovarian responders and in improving outcomes following frozen–thawed transplantation of autologous ovarian tissue. Further studies are required to explore the potential role of PRP in reproductive medicine further, to help standardise PRP protocols and evaluate which routes of administration are most effective.

## 1. Background

The use of cell-based therapies, such as platelet-rich plasma (PRP), has gained considerable momentum over the last decade due to their ability to promote tissue regeneration through cell differentiation and trophic activities. The first clinical application of PRP was as a transfusion product to treat thrombocytopenia [1]. It has since been used across numerous medical fields, including maxillofacial and plastic surgery, orthopaedic surgery, dermatology, urology and more recently gynaecology [2].

This review aims to summarise the use of PRP within the reproductive setting by conducting an evidence-based evaluation of its preparation, classification systems, mechanism of action and clinical applications. We aim to explore the potential benefits of PRP on endometrial receptivity and regeneration, embryo implantation and ovarian function.

PRP is an autologous plasma containing platelets prepared from fresh whole blood drawn from a peripheral vein. Through processing, it can be prepared to contain supraphysiological levels of platelets at three to five times greater than the level of normal plasma. Platelets are produced by megakaryocyte cells within the bone marrow. They are anucleate and have the smallest density amongst all blood cells with a diameter of 2 μL. Their physiologic count ranges from 150,000 to 400,000 platelets per μL [3]. Given their small density, centrifugation methods result in platelets settling at the top of an aggregate, which allows for efficient extraction and subsequent clinical use.

PRP contains alpha granules storing cytokines and growth factors, which are key to tissue regeneration [3,4]. Growth factors within the alpha granules include vascular endothelial growth factor (VEGF), transforming growth factor (TGF), platelet-derived growth factor (PDGF), epidermal growth factor (EGF), insulin-like growth factor-1 (IGF-1), connective tissue growth factor (CTGF) and fibroblast growth factor (FGF). These growth factors regulate cellular migration, differentiation and proliferation through autocrine and paracrine effects [5]. The alpha granules containing growth factors are released within ten minutes of platelet activation at the site of injury or inflammation, resulting in a net flow of neutrophils and macrophages, which leads to angiogenesis and re-epithelialisation [4,6]. Specifically within the human endometrial model, the release of PDGF has proven to be key to endometrial progenitor cell activity. PDGF isoforms have been demonstrated to significantly improve endometrial stromal cell proliferation and contractility [7].

The outer membrane of platelets consists of a phospholipid bilayer which provides the structural foundation of the platelet cell membrane. Phospholipids within the serum have been demonstrated to negatively correlate with the level of phospholipid within the follicular fluid within the ovarian follicles during controlled ovarian stimulation cycles in patients undergoing IVF [8]. This has been hypothesised to result from the increased consumption of platelets secondary to increased membranogenesis taking place during follicular growth. Therefore, an increased serum level of platelets through the addition of PRP, resulting in an increased level of serum phospholipid, may benefit follicular growth and thus improved oocyte during IVF cycles [9]. To add to this, Fayezi and colleagues discovered that the amounts of phospholipid and the phospholipid/apoA-I ratio in follicular fluid were associated negatively with the percentage of fertilised oocytes [10].

**Figure 1:**
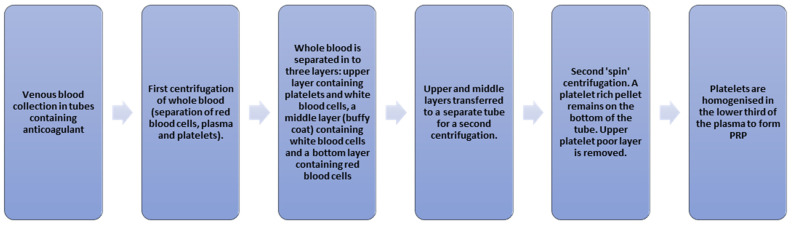
Steps involved in PRP formation.

The preparation of autologous PRP involves venepuncture and the collection of 20–30 mL of blood into sterile tubes containing an anticoagulant. This anticoagulant usually contains citrate and dextrose of sodium citrate to prevent the premature activation of platelets prior to use. The blood is centrifuged at moderate speed for several minutes at room temperature, which results in blood being separated into three layers according to the specific gravity of the components: the top clear-coloured supernatant layer, which is the platelet-rich plasma; the middle layer, known as the ‘buffy coat’, rich in white blood cells; and a bottom layer containing dense red blood cells. The PRP can then be collected and used. Figure 1 summarises the steps involved. PRP has been shown to remain viable for up to five days at room temperature [11]. A number of commercially available systems are available for the production of PRP, mostly varying in the volume of blood, the anticoagulant used and the speed and time of centrifugation required [12]. This does present a degree of heterogeneity amongst PRP products and, therefore, amongst studies evaluating the use of PRP, making cross-comparisons between different units less robust. There is currently no consensus or standardised protocol for the preparation of PRP, although the preparation of PRP is closely related to its clinical efficacy [13].

## 2. Types of PRP

The growth factors secreted by platelets which are available to tissues have been shown to be directly proportional to the platelet concentration. Bone and enhanced soft-tissue healing have been proven to occur at platelet concentrations of 1,000,000 plate-lets/μL within a 5mL volume of plasma; thus, this is often used as a working definition of therapeutic PRP [4]. In the presence of varying concentrations of platelets within a platelet gel, human endothelial cells have displayed induced proliferation, motility and enhanced invasiveness in the pattern of a bell-shaped distribution, whereby higher concentrations have resulted in a reversal of the processes [14]. The optimal concentration for platelet stimulation and proliferation was reported to be 1.25 × 106 and for angiogenesis, 1.5 × 106 platelets/mL [14].

The Dohan Ehrenfest classification (2009) proposed categorising the various platelet preparations into four categories, each determined by the number of leucocytes and the fibrin content [15]:

1 Pure platelet-rich plasma (P-PRP) or leucocyte-poor PRP is a preparation with absent or low levels of leucocytes and a low-density fibrin network following activation. The PRP products in this category can be used in liquid or in an activated gel form. It can therefore be used topically on skin wounds or over sutures;
2 Leucocyte and PRP preparations (L-PRP) contain leucocytes and a low-density fibrin network after activation. L-PRP products can also be used in a liquid or gel form. Similarly to P-PRP, it can also be spread over wounds or sutures;
3 Pure platelet-rich fibrin (P-PRF) is a preparation with absent or low levels of leucocytes and a high-density fibrin network. P-PRF products exist in an activated gel form and cannot be injected. Due to the fibrin matrix, it can be handled like a solid material. The fibrin matric provides a scaffold for cellular migration and tissue regeneration.

Leucocyte-rich fibrin and PRF (L-PRF) preparations are rich in leucocytes and a high-density fibrin network. The resultant clot or ‘biomaterial’ has a gel-like form ready for use. It can be used directly to fill a cavity to promote tissue regeneration and healing, mixed with a bone material for remodelling in orthopaedics or compressed into a membrane ready for application over surgical sites.

## 3. Methods

A literature search was performed using Medline, PubMed, EMBASE and the Cochrane Library to identify relevant papers up to 2023. The key terms used in combi-nation with “PRP” and "platelet-rich plasma” were “fertility”, “infertility”, “thin endometrium”, “Asherman’s”, “endometrial receptivity”, “endometrial thickness”, “recurrent implantation failure”, “premature ovarian failure”, “premature menopause” and “ovarian reserve”. The titles and abstracts of all retrieved articles were screened, and relevant papers were included within this review. Relevant articles not available in English were excluded from this review.

A summary of findings from each study is included in Table 1. 

## 4. Intrauterine Application of PRP

PRP has been explored both in vivo and ex vivo in the human endometrium model with regard to its ability to harness the intrinsic regenerative capacity of the endometrium. Chang et al. first demonstrated successful endometrial expansion (>7 mm thickness) at 48–72 h post-treatment in five women undergoing frozen embryo transfer cycles, leading to pregnancies in all five treated with intrauterine autologous PRP infusions [16]. These findings were corroborated by a further study of ten women treated with intrauterine PRP intrauterine infusions, all of whom displayed increased endometrial thickness, and five subsequently went on to achieve successful pregnancies [17].

### 4.1. Asherman’s Syndrome

Asherman’s syndrome is characterised by damage to the endometrial basal layer resulting in a deficiency of endometrial regeneration capacity and subsequent intrauterine adhesions [18]. These adhesions can cause menstrual irregularities, cyclical pain, abnormal placentation, recurrent miscarriage and infertility [19]. The cause of Asherman’s syndrome is usually iatrogenic, commonly following dilatation and curettage of the uterine cavity for miscarriage or termination of pregnancy [20]. Other causes include opening of the uterine cavity during a myomectomy procedure, hysteroscopic resection of submucosal fibroids, radiotherapy, uterine artery embolisation and chronic endometritis [21]. Previously, the therapeutic focus has been to remove the adhesions to improve fertility; however, adhesions often reform. Thus, emphasis on harnessing the endometrium’s capacity to regenerate is likely to improve outcomes [22]. Failure to adequately repair the damaged functional endometrium may be due to the loss of progenitor cells usually present in the basal endometrium. Additionally, endometrial progenitor stem cell activity is also known to be impaired in women with low circulating oestrogen or high levels of inflammatory cells [23,24].

In this setting, intrauterine injection of PRP has been shown to increase implantation sites and subsequently live birth rates through the reduced expression of fibrosis-related factors (Tgfb1, Timp1 and Col1a1) [25]. Intrauterine injection of human PRP into a murine model of Asherman’s syndrome resulted in an increase of 2.1 vs. 4.6 (*p* < 0.01) implantation sites in the untreated versus treated group. The live birth rate was 83% in the PRP-treated group and 0% in the untreated group. Administration of PRP has also been shown to increase proangiogenic factors and promote the migration of endometrial stromal cells into sites of uterine injury in mice [26]. In vitro studies in humans assessing the impact of PRP in Asherman’s syndrome remain sparce. Aghajanova et al. first demonstrated PRP infusions in the human model, where two patients with Asherman’s syndrome received intrauterine PRP introduced through a Wallace IUI catheter immediately following adhesiolysis. Both cases led to successful pregnancies, and one case demonstrated improved endometrial thickness following PRP treatment. Although PRP did not demonstrate endometrial growth in one case, as measured by endometrial thickness, the result of a successful conception and pregnancy despite a thin endometrium supports the notion of improved endometrial function post-PRP infusion [27]. In one clinical trial, fifteen patients with AS received 1ml of intrauterine PRP two days post hysteroscopic adhesiolysis rather than immediately due to concerns over the dilution of PRP with hysteroscopic fluid. Repeat hysteroscopy 8–10 weeks post-therapy failed to demonstrate any improvement in the reoccurrence of intrauterine adhesions and menstrual bleeding pattern compared to the control group [28]. In a similar trial of 15 patients with moderate to severe AS, 0.5–1 mL of PRP was infused immediately post-adhesiolysis, but no statistically significant difference was observed in the endometrial thickness or in the clinical and biochemical pregnancy rate [29]. In contrast, subendometrial injection of 5 mL of PRP in addition to coating the intrauterine lining with 5 mL of PRP gel immediately post-hysteroscopic adhesiolysis in 30 women with Asherman’s syndrome was shown to result in a reduced rate of adhesions reforming and resulted in a significant increase in the duration of menses [30]. It appears that the higher volume of PRP, the timing immediately post-adhesiolysis and the utilisation of the subendometrial route may have contributed to the superior outcomes seen in this study.

### 4.2. Endometritis

Chronic endometritis is defined as persistent inflammation of the endometrial mucosa caused by bacterial pathogens. Diagnosis of chronic endometritis is made through sampling of the endometrium at hysteroscopy and the presence of plasma cells within the endometrial stroma on histological analysis [31]. The level of proinflammatory cytokines interleukin-6, interleukin-1β and tumour necrosis factor α are increased in women with chronic endometritis, which may affect cell migration and proliferation [32]. It has been associated with repeat implantation failure and recurrent miscarriage [33,34,35]. Current treatment for chronic endometritis rests largely on oral antibiotics [32]. However, although antibiotic treatment has been shown to improve the implantation and clinical pregnancy rates, those with ongoing chronic endometritis may continue to experience fertility issues compared to women successfully treated [32,36]. Autologous PRP represents a novel treatment approach for chronic endometritis. One recent case study demonstrated a successful live birth following intrauterine infusion of PRP in a patient with a history of chronic endometritis and six failed embryo transfers. Microbiological and scanning electron microscopy analysis during a subsequent menstrual cycle following an intrauterine PRP infusion revealed no evidence of chronic endometritis [37]. In the bovine model in vitro, PRP has been shown to downregulate the expression of proinflammatory cytokines IL-1β, IL-8 and iNOS. Additionally, PRP has been shown to upregulate the expression of ER-α, ER-β and PR genes, which are vital for pregnancy [38]. Furthermore, in the equine model in vivo, intrauterine infusion of PRP displayed a reduction in the intrauterine inflammatory response as measured by the percentage of neutrophils in uterine cytology and the nitric oxide concentration within the uterine fluid [39]. In clinical settings such as endometritis where endometrial regeneration is impaired, PRP has been shown to increase the expression of matrix metalloproteinases (MMP) MMP3, MMP7 and MMP26 within the endometrial stromal fibroblasts and mesenchymal stem cells [40]. MMPs have been shown to be vital for successful wound healing—an important step in recovery from endometritis [41]. As provisional, albeit limited, outcomes from animal studies appear promising, there is a further need for well-designed studies in humans.

### 4.3. Refractory Endometrium

In each menstrual cycle, there exists a period of four to five ‘opportune’ days for the human embryo to implant when the endometrium remains receptive [42]. The endometrial microenvironment determines endometrial receptivity. This is governed by changes to the uterine luminal and glandular cells, decidualisation of the endometrial stroma and increased leukocyte activity [42]. Sonographic markers, such as endometrial thickness and uterine artery blood flow, have proven to have a high negative predictive value and a low positive predictive value for a receptive endometrium [43]. Nevertheless, studies have demonstrated an endometrial thickness of 7 mm and above to be optimal for implantation and to result in improved clinical pregnancy rates [44,45]. An endometrial thickness <7 mm, which is unresponsive to hormonal therapy, has been defined as a refractory endometrium and is associated with suboptimal fertility rates [46,47]. A severe deficiency in angiogenic-related markers has been demonstrated in patients with a refractory endometrium, specifically leukemia inhibitory factor [48], vascular endothelial growth factor (VEGF) and β 3 integrin [49]. Thus, given the proangiogenic potential of autologous PRP, its application in the setting of subfertility secondary to refractory endometrium presents an exciting opportunity for patients who are unresponsive to conventional treatment methods.

Another mechanism by which PRP has been shown to improve endometrial receptivity is by improvement in the endometrial immune environment, which has been long known to play an important role [50]. Six women previously treated for intrauterine adhesions received an intrauterine infusion of 1 mL of PRP. Mid-luteal endometrial samples in the PRP treatment group displayed significant reduction in CD8 T cells, Th1 and NK cells as compared to the control group of parous women with a normal uterine environment determined through hysteroscopy or ultrasound. Microbiota analysis revealed that the presence of bacillus, a proinflammatory species found in the endometrium, was also significantly less in the PRP-treated group. The ability of PRP to modulate the endometrial immune cells and microbiome is an important finding for women with refractory endometrium following adhesiolysis.

A cross-sectional study in patients with a refractory thin endometrium <7 mm demonstrated that subendometrial injection of autologous PRP under hysteroscopic guidance into the endomyometrial junctional zone across all four walls of the uterus resulted in a 75% success rate in achieving an endometrial thickness ≥7 mm [51]. These positive results support the notion of the mode of action of PRP in releasing growth factors into the site of administration, the endomyometrial zone, an area which has been associated with low levels of VEGF in women with implantation failure [52]. VEGF has also been shown to improve vascular permeability in the mid-luteal phase, which has been proven to be essential for successful implantation of the embryo [52]. In one randomised controlled trial of 97 patients with repeated implantation failure, autologous PRP was infused directly into the uterine cavity with an embryo catheter 48 hours prior to embryo transfer. The PRP-treated group demonstrated a higher clinical pregnancy rate (44.89% versus 16.66%, *p*-value = 0.003) [53]. More specifically, in a cohort of 60 patients with a thin endometrium measuring <7 mm, the PRP-treated group demonstrated endometrial expansion to >7 mm following a second intrauterine infusion, a thickness which was not achieved in the placebo arm (7.21 ± 0.18 vs. 5.76 ± 0.97 mm, *p* = <0.001) [54]. The administration method included the infusion of 0.5 mL of PRP into the uterine cavity with a standard intrauterine insemination catheter. The second intrauterine infusion was administered 48 hours later. Interestingly, another study demonstrated increased Doppler flow signal to the endometrium post-intrauterine PRP treatment in women who had a pretreatment suboptimal endometrium (<7 mm) and suboptimal vascularity (<5 vascular signals) [55], suggesting PRP may also play a role in neovascularisation. Endometrial vascularity has been shown to be an important parameter in the implantation potential of the human endometrium [56]. Whilst there is significant heterogeneity amongst studies so far on the route of application for the intrauterine delivery of PRP, an observational study comparing the subendometrial and intrauterine infusion routes did not demonstrate a significant difference in clinical pregnancy rates (51% vs. 52.3%) [57].

## 5. Intraovarian Injection of PRP

### 5.1. Premature Ovarian Insufficiency

Premature ovarian insufficiency (POI) is a devastating diagnosis for women who have not yet met their reproductive aspirations. It is characterised by menopausal levels of gonadotrophin follicle-stimulating hormone, sex steroid deficiency and follicular atresia leading to absent or irregular menstrual cycles prior to the age of 40 years [58]. Intermittent ovarian function is observed in some patients with POI, and in almost 1 in 4 patients, resumption of ovarian function is observed, and spontaneous pregnancies have been reported [59,60]. This offers the hope of therapeutic reversal of ovarian atresia by harnessing the potential of PRP to promote the growth of primordial and preantral follicles. One example is the outcome from in vitro studies which have found PRP-cultured media yield significantly higher rates of growth and survival in human preantral follicles when compared to standard protocols [61].

The first human in vivo study to report the intraovarian injection technique described a case series of four women with POI who underwent intraovarian injection of 5 mL of autologous PRP under ultrasound guidance [62]. A decrease in follicular-stimulating hormone (FSH) and an increase in anti-Mullerian hormone (AMH) was observed in all cases. Moreover, each patient yielded 4–7 oocytes and at least 1 blastocyst. One possible explanation for the successes observed in this setting is the presence of latent oocytes which have responded to PRP-induced growth factors. Alternatively, PRP growth signalling pathways may have helped to support the ovarian niche to induce the development of pluripotent ovarian stem cells into germ cells. Persuasive evidence does exist in support of the capability of ovarian germ cells to generate de novo oocytes [63,64]. Ovarian folliculogenesis is a complex process. The preantral phase is governed by the ovary, with the secretion of local growth factors via autocrine and paracrine mechanisms. The second phase, which proceeds to either ovulation or atresia, is gonadotrophin dependent. The autocrine and paracrine mechanisms are regulated via growth factors, including bone morphogenic proteins (BMPs), growth differentiation factors (GDFs), TGF-β, activins and inhibins and anti-Mullerian hormone (AMH). The expansion of cumulus cells is crucial to folliculogenesis and is hypothesised to be supported by oocyte-produced growth and differentiation factor 9 (GDF9) [65]. Thus, these ovary-dependent stages maintain the intrinsic capabilities of the ovary and support the notion of the ovary as a suitable direct target for therapeutic intervention with PRP. PRP has been hypothesised to stimulate neoangiogenesis within the ovary through the introduction of PRP-induced growth factors.

In murine models of POI, intraovarian PRP injection has been shown to influence the genes involved in angiogenesis possibly playing a role in ovarian restoration [66]. Statistically higher expression of transcripts of ANGPT2 and KDR were noted in both the low- and high-concentrate PRP groups when compared to controls, with the highest levels at week six following injection. In PRP-treated POI rats, morphologically normal follicular counts were restored at almost all stages of follicular development. This may support the notion of the involvement of proangiogenic pathways for ovarian tissue restoration.

In a large observational study, 469 women with a history of infertility underwent intraovarian injection of 2–4 mL of PRP into each ovary. A significant decrease in FSH and increase in oestradiol across all age groups (32–46 years) was reported [67]. However, despite holding power on the largest sample size to date, the study included a mixed cohort of women with primary amenorrhoea, POI and hormonal abnormalities. Subgroup analysis was not performed, thus making the role of PRP unclear in this setting.

Cakiroglu et al. recruited 311 women with POI (diagnosed as per the European Society of Human Reproduction and Embryology [ESHRE] criteria) to undergo intraovarian PRP injection [68]. A total of 2–4 mL of PRP was injected into multiple sites within the ovaries. Timing of the injection was random in the amenorrhoeic group, and in the oligomenorrheic group, the injection took place on the tenth day following cessation of menstrual bleeding. Six weeks of expectant management followed to allow for spontaneous pregnancy, which occurred in 23 patients (7.4%). Amongst the remaining 201 women, 70% developed at least one antral follicle following PRP treatment. Of those who were suitable for oocyte retrieval, the mean number of oocytes per retrieval was 1.8 ± 1.3. In total, 41% of women who underwent stimulation achieved at least one cleavage stage embryo. The study highlights the potential of PRP in women with POI. Interestingly, women who had at least one antral follicle present at the time of PRP injection were more likely to respond, suggesting that PRP plays a role in the activation, growth and differentiation of the follicle.

Mechanical disruption of the ovarian cortex by performing an ovarian scratch laparoscopically has been hypothesised to manipulate activation and growth of dormant primordial follicles [69]. It is, therefore, unclear whether the injection of PRP into the ovarian cortex is directly linked to the action of PRP or the impact of the injection.

### 5.2. Embryo Quality

Intraovarian injection of PRP before commencing IVF has been shown to have a positive effect on the embryo euploidy rate [70]. Twelve women with a history of one failed IVF cycle, had approximately 4 mL of PRP injected through multiple punctures into the subcortical layers of each ovary. The euploid embryo number increased from 8% in the untreated cycle to 39% in the cycle where PRP was administered prior to starting, within a three-month timeframe. The lack of a control group undergoing ovarian puncture only, without PRP injection, remains a limitation of this study.

### 5.3. Menopause and Ovarian Ageing

Age-related reproductive decline leaves women with no option but to explore the possibility of having non-genetically related offspring utilising oocyte donation. Autologous platelet-rich plasma presents a possible option to reverse this reproductive predicament.

Intraovarian injection of autologous PRP in a series of three menopausal women proved successful in achieving an ongoing clinical pregnancy beyond the second trimester across all three cases [71]. In this series, three women aged 46, 40 and 27 years with a diagnosis of menopause had 4 mL of PRP injected into each ovary. Each sample contained approximately 250,000 µ/L of platelet concentrate. The authors highlight the issue of leakage from the atrophic menopausal ovary, which was observed and emphasises the challenge of standardising the PRP volume in future studies. Following PRP injection, menstrual cycles resumed at 1.2 months and 1 month, respectively, folliculogenesis was observed and natural conception was achieved between 2 and 6 months. The study does add value to the merits of intraovarian PRP injection in restoring fertility following menopause; however, a direct cause and effect for why menstrual cycles resumed in this cohort following PRP injection cannot be concluded.

In the murine model of ovarian ageing, a single injection with a combination of bone marrow-derived stem cells and PRP presented a synergistic effect on improving the growth of primary follicles in the older cohort [72]. A significant reduction in the number of fragmented oocytes and an increase in the number of oocytes entering metaphase II was observed in addition to improved chromosomal alignment. The older group receiving PRP and stem cells also displayed improvements in the blastocyst formation rate. Improved morphologic appearances were matched with reduced mitochondrial dysfunction, improved mitochondrial copy numbers and a reduction in oxidative damage. Thus, ovarian recovery has been demonstrated; however, human studies are required to confirm these findings. In vivo human studies are needed to demonstrate the interaction of functional ovarian recovery, implantation and the continuation of a successful clinical pregnancy.

### 5.4. Ovarian Cortex Transplantation

Frozen–thawed transplanted autologous ovarian tissue has been shown to be successful in restoring fertility, but in many cases the autograft suffers ischaemia, which results in a subsequent loss of follicles until neoangiogenesis is restored [73]. Callejo et al. performed an ovarian cortex transplant, which was injected and coated with PRP gel, that subsequently led to a successful live birth [74]. The PRP was prepared by taking 60 mL of the patient’s own blood and centrifuging it twice to obtain a platelet pellet immersed in 5 mL of plasma. The FSH reduced from over 40 mIU/mL to 7.9 mIU/mL, and the oestradiol level increased accordingly from 58 pg/mL to 316 pg/mL, suggestive of the resumption of hormonal activity. Clinically, whilst the follicles only reached a maximum of 14 mm after nine days of stimulation, the decision to trigger was based upon serum oestradiol levels. Following an ovulation trigger with human chorionic gonadotrophin, two oocytes were obtained, one in metaphase II and the other in metaphase I initially, then transitioning to metaphase II after seven hours of culture. For oncological patients, the transplantation of cryopreserved ovarian tissue upon achieving remission, despite its success, may not be an option due to the risk or reintroducing malignant cells [75]. Thus, in vitro folliculogenesis represents a vital clinical alternative. Optimisation of the growth of preantral stage follicles to the antral stage remains an important step in improving the success rate of in vitro folliculogenesis. In vitro, culture media supplemented with PRP was shown to support the growth and survival of early preantral follicles significantly better than media without PRP [61]. In comparison to other growth supplements routinely used in culture media, such as human serum albumin and fetal bovine serum, human platelet lysate (hPL), a derivative of PRP, has demonstrated improvement in follicular growth and survival of isolated primary and secondary human ovarian follicles [76].

### 5.5. Suboptimal Ovarian Response in In Vitro Fertilisation

In the setting of poor ovarian response [POR], where there is a reduction in quantity and quality of oocytes, intraovarian injection of PRP has been utilised to improve AMH and reduce FSH levels in addition to increasing the number of oocytes retrieved [73,77]. Sfakianoudis et al. reported a case series where 5 mL of PRP was injected into the ovaries of three women aged 37–40 years, who were poor responders to standard stimulation protocols. A 75% improvement in AMH and a 67% decrease in FSH levels was observed within three months of the treatment. Clinical pregnancies were achieved in all three patients, with one ongoing pregnancy and two reported live births. (77) In the poor ovarian reserve cohort, a significant increase in AMH was observed in 86% of women following intraovarian PRP injection [78]. Interestingly, age, body mass index and the duration of infertility did not correlate with a response to intraovarian PRP injection.

In a large observational study, 510 patients aged between 30 to 45 years with a diagnosis of POR using the POSIEDEN criteria underwent intraovarian injection of PRP [79]. A volume of 2–4 mL of autologous PRP was injected into one or both ovaries via the transvaginal route using ultrasound guidance during the follicular phase of the menstrual cycle. Spontaneous pregnancy occurred in 4.3% of patients during the expectant management period. A total of 474 patients underwent controlled ovarian stimulation (COS). The mean number of oocytes per retrieval before and after PRP treatment were 2.2 ± 1.9 and 3.4 ± 2.7 (*p* < 0.001), respectively. Following PRP treatment, 66% of women who underwent COS achieved at least one day 3–5 embryo. The mean number of blastocysts obtained before and after PRP treatment were 0.6 ± 0.9 and 2.3 ± 1.6 (*p* < 0.001) The cohort demonstrated a pregnancy rate of 20.5% and a combined implantation and live birth rate of 12.9%. The volume of PRP injected did not correlate with the outcome.

### 5.6. Strengths and Limitations

This review provides a valuable overview of the clinical application of PRP for female patients within the reproductive setting. A wide range of study designs has been included, from case series to case control studies, which have provided a broad summary. It is currently the most comprehensive overview of the topic and will help to guide future research directions. Due to the lack of randomised controlled trials on the topic, a narrative review was selected as the research methodology. However, narrative reviews can introduce bias in the articles selected due to the lack of standardised methodology for data extraction, as is the case with systematic reviews. The authors recommend a systematic review and meta-analysis when further randomised controlled trials are available.

## 6. Conclusions

**Figure 2:**
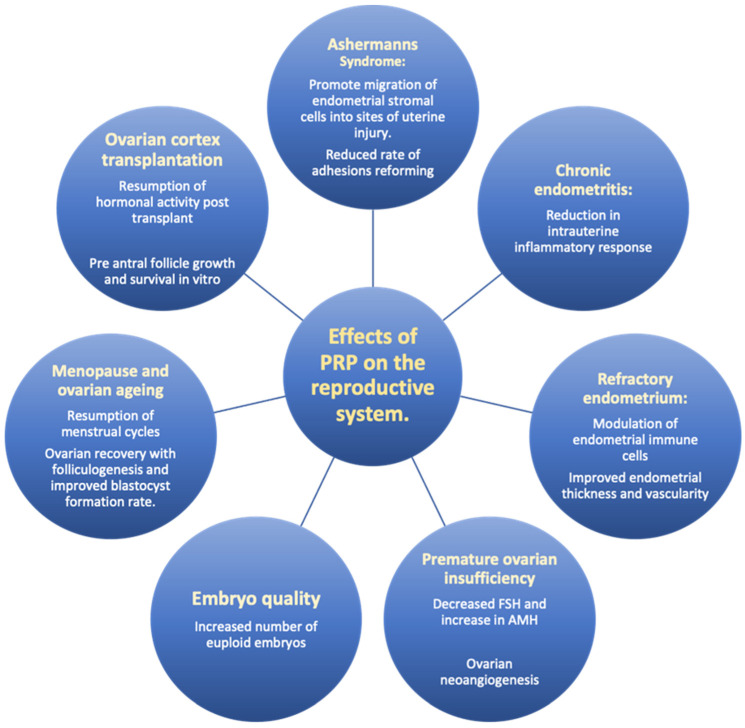
Effects of PRP on the reproductive system.

Intrauterine infusion of PRP represents a novel strategy for the treatment of the endometrium in its ability to promote biological processes for endometrial regeneration. Data on the benefit of PRP within the reproductive setting still remain scarce. However, the theoretical benefits and positive preliminary findings suggest great potential in other indications within reproductive medicine (Figure 2). PRP offers an exciting opportunity to enhance ovarian reserve in the context of POI, poor ovarian response and potentially in the context of ovarian cortex transplantation. PRP remains a relatively low-cost therapeutic intervention given that it can be prepared at the patient’s bedside with minimal equipment and can be administered in the office setting quickly and effectively. The use of autologous blood to produce PRP has eliminated the risk of immunological reactions and presents a widespread opportunity for its use in the field of gynaecology. However, differences in PRP preparation can produce a heterogenous injectate, which may vary in quality, purity and quantity. One such example is the variation of centrifugation speed and duration where higher speeds can result in a greater concentration of platelets but may also result in more contaminants or disruption in the platelet integrity. Future studies should define the cellular content of PRP, including the white and red cell counts, the concentration factor and the platelet yield—a valuable step in producing a robust product for clinical application. Moreover, further studies need to evaluate the optimal methods and routes of administration. Despite the clear potential of the role of PRP in reproductive medicine, well-designed, randomised, prospective studies are essential before usage can be recommended. One particularly valuable area of focus is the effect of PRP in the activation and growth of ovarian follicles in addition to the potential for reversal of ovarian ageing.

## Figures and Tables

**Table 1:** Summary of studies utilising PRP for infertility treatments.

Population	Author	Year	Sample Size	Study Design	Model	Route of PRP Injection	Volume of PRP Injected	Main Findings following PRP Therapy
Asherman’s	Kim et al.	2020	35	Case control	Murine	Intrauterine	0.7 mL	Decreased expression of fibrosis-related factors following endometrial injury, enhanced number of implantation sites, improvement in the live birth rates in mice with AS.
Asherman’s	Kim et al.	2022	5	Case control	Murine	Intrauterine	n/a	Increased the mRNA expression levels of proangiogenic factors. Significantly higher number of implantation sites in treated group.
Asherman’s	Aghajanova et al.	2018	2	Case study	Human	Intrauterine	n/a	Improved endometrial thickness and function. Improved pregnancy rate.
Asherman’s	Javaheri et al.	2020	30	Nonrandomised clinical trial	Human	Intrauterine	1 mL	No improvement in the reoccurrence of intrauterine adhesions. No difference in the menstrual bleeding pattern compared to the control group.
Asherman’s	Aghajanova et al.	2021	30	RCT	Human	Intrauterine	0.5–1 mL	No statistically significant difference observed in the endometrial thickness nor the clinical and biochemical pregnancy rate.
Asherman’s	Amer et al.	2018	60	RCT	Human	Subendometrial	5 mL	Reduced rate of adhesions reforming. Significant increase in the duration of menses.
Endometritis	Sfkianoudis et al.	2019	1	Case report	Human	Intrauterine	2.5 mL	No microbiological evidence of chronic endometritis following treatment. Successful live birth.
Endometritis	Reghini et al.	2016	21	RCT crossover	Mare	Intrauterine	20 mL	Reduction in percentage of neutrophils.
Refractory endometrium	Chang et al.	2023	12	Case control	Human	Intrauterine	1 mL	Improved endometrial receptivity and significantly lower level of endometrial NK cells, CD8 T cells and Th1 cells.
Refractory endometrium	Agarwal et al.	2020	32	Cross-sectional	Human	Subendometrial	4 mL	A success rate of 75% in achieving an endometrial thickness greater than 7 mm.
RIF	Nazari et al.	2019	97	RCT	Human	Intrauterine	0.5 mL	Higher clinical pregnancy rate (45% versus 17%).
Refractory endometrium	Nazari et al.	2019	60	RCT	Human	Intrauterine	0.5 mL	Endometrial expansion to >7 mm following a second intrauterine infusion. Biochemical pregnancy in 12/30 treated patients.
Refractory endometrium and RIF	Tandulwadkar et al.	2017	68	Observational	Human	Intrauterine	0.5–0.8 mL	Endometrial expansion to >7 mm, significant increase in endometrial vascularity, 61% implantation rate, 45% clinical pregnancy rate.
RIF	Noushin et al.	2021	318	Observational	Human	Intrauterine and Subendometrial	n/a	PRP-treated group demonstrated a higher LBR overall, no difference in outcomes between intrauterine and subendometrial injection.
POI	Sills et al.	2018	4	Case series	Human	Intraovarian	5 mL per ovary	Improved ovarian function as early as two months after treatment. All four patients achieved blastocysts.
POI	Ahmadian et al.	2020	86	Case control	Murine	Intraovarian	0.1 mL per ovary	Both high and low PRP concentrations displayed improvement in follicular quality, statistically significant increase in number of morphologically normal follicles when compared to the controls.
POI	Fraidakis et al.	2023	469	Observational	Human	Intraovarian	2–4 mL per ovary	Significant improvement in FSH and oestrodiol levels.
POI	Cakiroglu et al.	2020	311	Observational	Human	Intraovarian	2–4 mL per ovary	Significant increase in AFC and serum AMH, 7% spontaneous conception. In total, 41% of patients undergoing stimulation achieved at least one embryo.
POI	Pantos et al.	2019	3	Case series	Human	Intraovarian	4 mL per ovary	Decreased FSH and increase in AMH across all cases. Menstrual cycle restoration. Clinical pregnancy through natural conception in all cases.
POI	Marchante et al.	2023	36	RCT	Murine	Intraovarian	0.1 mL per ovary	Bone marrow–derived stem cells combined with PRP promoted follicle maturation across mice of all age groups. In the mature group, treatment led to improvement in the quantity and quality of ovulated mature oocytes.
Frozen thawed ovarian tissue	Callejo et al.	2013	1	Case study	Human	Ovarian tissue coating	n/a	Resumption of ovarian hormonal activity and ovarian follicle growth to 15 mm. Live birth.
POR	Sfkianoudis et al.	2019	3	Case series	Human	Intraovarian	5 mL per ovary	A 75% improvement in AMH, 67% decrease in FSH levels within three months of treatment. Clinical pregnancies were achieved in all three patients.
POR	Hosseinisadat et al.	2023	22	Observational	Human	Intraovarian	n/a	Significant increase in AMH in 86% of women.
POR	Cakiroglu et al.	2022	510	Observational	Human	Intraovarian	2–4 mL per ovary	In total, 66% of women who underwent COS achieved at least one day 3–5 embryo. There was a 21% pregnancy rate and a combined implantation and live birth rate of 13%.

Key—RCT: randomised controlled trial, RIF: recurrent implantation failure, POI: premature ovarian insufficiency, AFC: antral follicle count, AMH: anti-Mullerian hormone, FSH: follicle stimulating hormone, POR: poor ovarian responder, COS: controlled ovarian stimulation.

## Data Availability

Data sharing not applicable. No new data were created or analyzed in this study. Data sharing is not applicable to this article.
